# Boosting circadian autophagy by means of intermittent time-restricted feeding: a novel anti-ageing strategy?

**DOI:** 10.20517/jca.2021.33

**Published:** 2022-01-01

**Authors:** Sebastiano Sciarretta, Maurizio Forte, Junichi Sadoshima

**Affiliations:** 1 Department of Medical and Surgical Sciences and Biotechnologies, Sapienza University of Rome, Latina 04100, Italy.; 2 Department of AngioCardioNeurology, IRCCS Neuromed, Pozzilli 86077, Italy.; 3 Department of Cell Biology and Molecular Medicine, Rutgers New Jersey Medical School, Newark, NJ 07103, USA.

The increase in life expectancy due to medical advances in the treatment of human diseases is often paralleled by an accumulation of age-related complications that progressively compromise the function of fundamental organs such as skeletal muscles, bones, heart, and brain, and may, in many cases, lead to permanent disabilities. Therefore, a major challenge for scientists in the future is to find new strategies to delay age-related complications. In order to achieve this goal, it is essential to fully characterize the major molecular changes occurring in tissues and organs during the ageing process, contributing to its progression and deleterious effects. These strategies should be aimed not only at extending lifespan but also at extending healthspan by reducing ageing-related morbidity.

In recent years, autophagy has emerged as a promising anti-ageing mechanism^[1]^. Autophagy is an intracellular evolutionarily conserved catabolic mechanism devoted to the removal of senescent and damaged cytoplasmic elements, including cellular organelles. Constituents of digested elements are then reused by the cell for energy production, new molecule synthesis, or other metabolic processes. The activation of autophagy in response to stress preserves cell energy, reduces oxidative stress, attenuates misfolded protein accumulation, and guarantees mitochondrial turnover, thereby ensuring cell survival. A large body of evidence have suggested that autophagy declines during ageing, compromising organ functions due to the accumulation of misfolded proteins and dysfunctional organelles^[2]^. In pre-clinical models of ageing, restoration of cardiac autophagy was reported to improve cardiac function and to increase lifespan^[2]^. Similarly, autophagy activation has been shown to be beneficial in neurodegenerative diseases^[1]^.

To date, several dietary strategies to rescue autophagy during ageing have been tested. Most of these approaches involve reducing nutrient intake, since fasting is efficacious in stimulating autophagy. For example, calorie restriction (CR), defined as the reduction of calorie intake without malnutrition, is a potent autophagy inducer and was reported to preserve organ function, including cardiac performance, in aged rodents^[3]^. Alternatively, several synthetic and natural compounds able to mimic the beneficial effects of CR, called calorie restriction mimetics (CRMs), were shown to delay ageing and to increase lifespan in several species, from yeast to primates^[3]^. For most of the CRMs, the anti-ageing effects were mediated by autophagy activation^[3]^. The natural polyamine, spermidine, is a well characterized CRM. Spermidine extends lifespan in mice and reduces cardiac ageing, and these effects are abrogated in mice with cardiac-specific deletion of autophagy related 7 (ATG7), a fundamental component of autophagy, suggesting a mechanistic link between autophagy and spermidine-induced lifespan extension^[4]^. Epidemiological studies also revealed that spermidine intake reduces cardiovascular death and increases longevity in human subjects^[4]^. At the molecular level, CRMs reduce the acetylation state of cellular proteins, with the net result of activating autophagy through stimulation of adenosine monophosphate-activated protein kinase and inhibition of mammalian target of rapamycin complex 1 signalling pathways^[3]^.

Another approach able to increasing longevity is time-restricted feeding (TRF), defined as the restriction of food consumption to certain hours of the day (usually less than 12 h)^[5]^. By reprogramming circadian rhythms, TRF was reported to exert pleiotropic effects in preclinical models, reducing metabolic diseases in mice and improving cardiac function in *Drosophila*, for example^[5]^. In human subjects, TRF was found to improve metabolic health, physical endurance, and blood pressure, especially in overweight individuals^[6]^. However, the molecular mechanisms by which TRF exerts its protective effects remain unclear. In a recent paper published in *Nature*, Ulgherait *et al*.^[7]^, after several attempts, developed a TRF protocol, named intermittent TRF (iTRF), able to robustly and consistently extend lifespan in *Drosophila*. This new dietary protocol was a night-biased iTRF and consisted of 20-h fasting every other day (starting at mid-morning), with a recovery day of *ad libitum* diet between fast days [Figure 1]. The authors observed that 30 days of iTRF, from day 10 to day 40 of age, extends lifespan and improves functional capacities in flies. Unlike with other dietary restriction regimens, Ulgherait *et al*.^[7]^ observed that iTRF-induced longevity is independent of calorie restriction, reduction of protein intake, or inhibition of insulin-like signalling. In fact, flies undergoing iTRF combined with dietary protein restriction or inhibition of insulin-like signalling showed a significant additional increase in longevity, similar to the percentage of lifespan increase observed in iTRF alone versus control groups. These results suggest that these interventions act through distinct mechanisms and may be combined to achieve an enhanced effect. Ulgherait *et al*.^[7]^ also observed a reduction in markers of ageing in the gut and muscles of *Drosophila*, and an increase in climbing activity, indicating that iTRF not only increased lifespan, but was also associated with increased healthspan.

In order to explore the molecular mechanisms by which iTRF induces longevity, the circadian clock components were investigated^[7]^. iTRF enhanced night-time expression of the circadian clock components period (*per*) and timeless (*tim*), and iTRF-mediated lifespan extension required a functional circadian clock. In fact, iTRF did not induce lifespan extension in circadian mutants. The increased expression of clock genes in response to iTRF was found to lead to increased night-time activation of autophagy through the expression of autophagy related 1 (*atg1*) and autophagy related 8 (*atg8*) (homologues of mammalian ULK1 and LC3, respectively), two important autophagy genes that promote autophagosome formation. Knockdown of *atg1* or *atg8* abrogates the effects of iTRF on lifespan extension, suggesting that autophagy is the molecular mechanism by which iTRF induces longevity. Remarkably, genetic or pharmacological nightinduction of autophagy was also found to extend lifespan in flies with continuous access to food (*ad libitum*), without showing additive effects upon lifespan extension with respect to iTRF. In contrast, when autophagy was upregulated during the day, no increase in lifespan was observed^[7]^.

Overall, the results obtained by Ulgherait *et al*.^[7]^ indicate that circadian-regulated induction of autophagy is not only required for the lifespan and healthspan extending effects of iTFR but is also sufficient to significantly attenuate the ageing process independently of a specific dietary protocol. These data extend previous evidence regarding the role of autophagy as a fundamental anti-ageing mechanism, suggesting that continuous activation of autophagy may be less effective than pulsed stimulation entrained with the circadian rhythm. Night-time administration of autophagy activators may be an option. Compounds able to boost autophagy with a circadian rhythm should also be developed, particularly for conditions like obesity and nutrition overload, which were previously found to disrupt circadian regulation of fundamental metabolic processes in the heart, leading to cardiac remodelling^[8]^. Pharmacological regulators of circadian rhythm machinery may be effective for this purpose. Alternatively, iTRF may represent a valid strategy to boost autophagy with a circadian rhythm and without restriction of food intake. This approach seems very feasible, since activity is usually lower during the night. However, important aspects regarding this topic remain to be clarified in the future. It will be important to investigate whether a similar iTRF protocol is able to extend lifespan in mammals, and if so, whether these effects are mediated by the regulation of circadian rhythm machinery and stimulation of autophagy. In this regard, the anti-ageing effects of iTRF may vary from organ to organ, since previous work suggested that the effects of TRF on circadian rhythm are tissue-specific, with liver and fat being sensitive to circadian rhythm entrainment by TRF, whereas heart and kidney are resistant^[6]^. Future studies are required to understand the molecular pathways through which TRF regulates the circadian rhythm machinery in a tissue-specific manner. Interestingly, previous work demonstrated that intermittent fasting, which shared some similarities with TRF, reduced cardiac ischemia/reperfusion injury in mice through autophagy stimulation. It would be interesting to evaluate whether the beneficial effects of intermittent fasting in the heart, including autophagy activation, are dependent on circadian rhythm modulation^[9]^. The molecular mechanisms by which circadian autophagy stimulation induces anti-ageing effects should also be clarified. In this regard, the circadian core regulatory gene *Clock* was recently reported to preserve cardiomyocyte survival following ischemia, through the improvement of mitochondrial quality control mechanisms, such as mitochondrial dynamics and mitophagy^[10]^, thus representing a valid strategy to reduce cardiovascular risk in subjects with circadian disorders. It will be interesting to test whether iTRF is able to attenuate ageing by stimulating mitophagy and mitochondrial dynamics^[10]^. Finally, the effects of iTRF on specific diseases occurring during the progression of ageing should also be tested in future studies in mouse models of diseases, particularly in those conditions associated with autophagy impairment. It will be important to evaluate whether iTRF is able to reduce cancer development, attenuate cardiovascular damage during different stresses, or prevent the development and progression of neurodegenerative disorders, and, if so, whether these effects are mediated by autophagy activation.

In conclusion, if confirmed in other experimental models, these recent findings suggest that iTRF can be considered a new beneficial lifestyle intervention to increase longevity and improve healthspan by optimizing circadian rhythm and stimulating circadian autophagy.

## Figures and Tables

**Figure 1.**
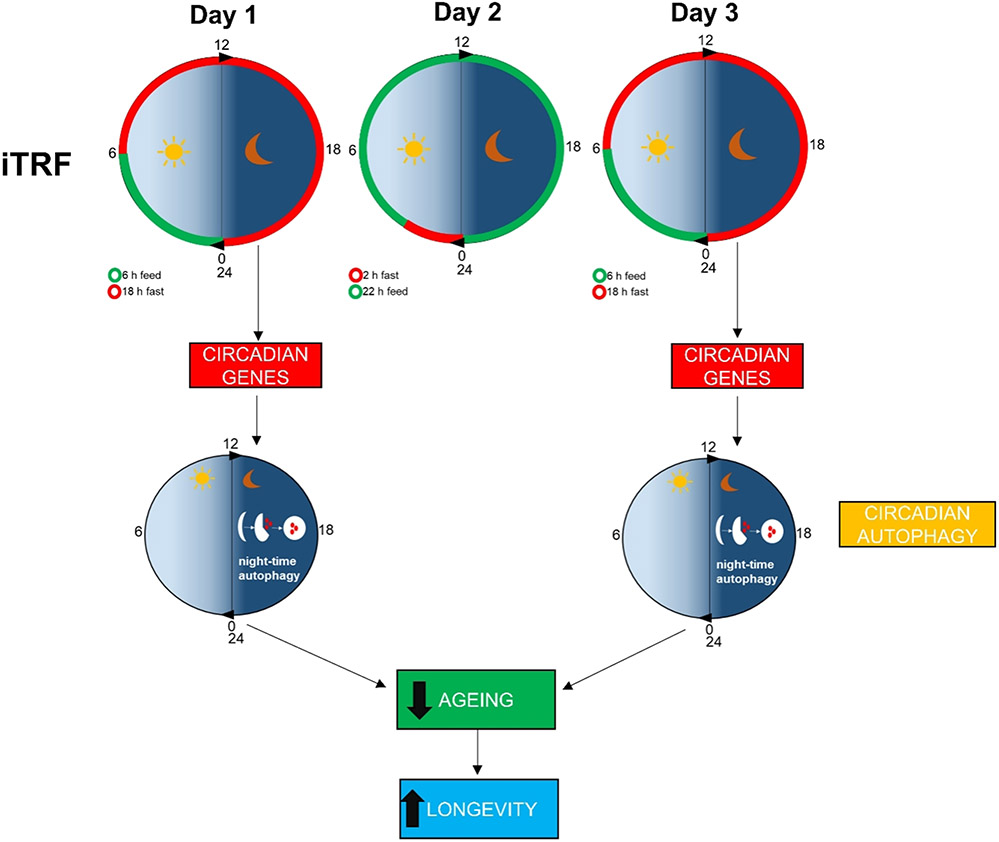
iTRF-induced increase of longevity is mediated by circadian induction of autophagy. Schematic representation of iTRF schedule (top panel). iTRF induces night-time overexpression of autophagic genes. Circadian upregulation of autophagy reduces ageing, leading to increased longevity. iTRF: intermittent TRF.
